# Genomic Characterization of Carbapenemase-Producing *Enterobacter hormaechei*, *Serratia marcescens*, *Citrobacter freundii*, *Providencia stuartii*, and *Morganella morganii* Clinical Isolates from Bulgaria

**DOI:** 10.3390/antibiotics13050455

**Published:** 2024-05-16

**Authors:** Stefana Sabtcheva, Ivan Stoikov, Ivan N. Ivanov, Deyan Donchev, Magdalena Lesseva, Sylvia Georgieva, Deana Teneva, Elina Dobreva, Iva Christova

**Affiliations:** 1 Laboratory for Clinical Microbiology, National Oncology Center, 1797 Sofia, Bulgaria; istoykov@sbaloncology.bg (I.S.); sigeorgieva@sbaloncology.bg (S.G.); 2 Department of Microbiology, National Center of Infectious and Parasitic Diseases, 1504 Sofia, Bulgaria; iivanov@ncipd.org (I.N.I.); deyandonchev@ncipd.org (D.D.); d.teneva@ncipd.org (D.T.); elina_dobreva@ncipd.org (E.D.); iva_christova@ncipd.org (I.C.); 3 Department of Microbiology, University Multiprofile Hospital for Active Treatment and Emergency Medicine “N. I. Pirogov”, 1606 Sofia, Bulgaria; magdalena.leseva@pirogov.bg

**Keywords:** carbapenemases, NDM-1, VIM-4, VIM-86, OXA-48, horizontal gene transfer, *Enterobacter*, *Serratia*, *Citrobacter*, *Providencia*, *Morganella*

## Abstract

Carbapenemase-producing *Enterobacter* spp. *Serratia marcescens*, *Citrobacter freundii*, *Providencia* spp., and *Morganella morganii* (CP-ESCPM) are increasingly identified as causative agents of nosocomial infections but are still not under systematic genomic surveillance. In this study, using a combination of whole-genome sequencing and conjugation experiments, we sought to elucidate the genomic characteristics and transferability of resistance genes in clinical CP-ESCPM isolates from Bulgaria. Among the 36 sequenced isolates, NDM-1 (12/36), VIM-4 (11/36), VIM-86 (8/36), and OXA-48 (7/36) carbapenemases were identified; two isolates carried both NDM-1 and VIM-86. The majority of carbapenemase genes were found on self-conjugative plasmids. IncL plasmids were responsible for the spread of OXA-48 among *E. hormaechei*, *C. freundii*, and *S. marcescens*. IncM2 plasmids were generally associated with the spread of NDM-1 in *C. freundii* and *S. marcescens*, and also of VIM-4 in *C. freundii*. IncC plasmids were involved in the spread of the recently described VIM-86 in *P. stuartii* isolates. IncC plasmids carrying *bla*_NDM-1_ and *bla*_VIM-86_ were observed too. *bla*_NDM-1_ was also detected on IncX3 in *S. marcescens* and on IncT plasmid in *M. morganii*. The significant resistance transfer rates we observed highlight the role of the ESCPM group as a reservoir of resistance determinants and stress the need for strengthening infection control measures.

## 1. Introduction

*Enterobacterales* such as *Enterobacter* spp., *Serratia marcescens*, *Citrobacter freundii*, *Providencia* spp., and *Morganella morganii* (ESCPM group) are increasingly identified as causative agents of nosocomial infections, frequently including bloodstream, urinary tract, gastrointestinal, respiratory, and skin and soft tissue infections [1,2]. These microorganisms produce chromosomally encoded inducible AmpC β-lactamase, belonging to Ambler class C and Bush group 1 [3]. Low-level expression of this enzyme is sufficient for the hydrolysis of aminopenicillins, aminopenicillin/β-lactamase inhibitor combinations, and first-generation cephalosporins. However, AmpC overproduction can occur following exposure to β-lactams, either through induction or selection of derepressed mutants during therapy, which leads to the hydrolysis of third-generation cephalosporins as well. As a result, the use of last-resort antibiotics such as carbapenems may become imperative in managing infections caused by AmpC-producing organisms [4]. Moreover, the intrinsic colistin resistance inherent to *S. marcescens*, *M. morganii*, and *Providencia* spp. further limits therapeutic options and contributes to their growing prevalence [5,6,7].

On the other hand, the widespread use of carbapenems as the preferred therapy for multiresistant ESCPM infections carries the risk of promoting carbapenem resistance. Such resistance can arise from various mechanisms, including diminished outer membrane permeability due to reduced expression or inactivation of outer membrane porins, mutations resulting in increased efflux and alterations in the structure of penicillin-binding proteins, or a combination of AmpC overexpression and extended spectrum of β-lactamase (ESBL) with porin mutations. However, the most concerning scenario is the acquisition of carbapenemases [8,9,10].

In Enterobacterales, five prevalent carbapenemase families have been identified across three Ambler classes: KPC (class A); IMP, NDM, and VIM (class B); and OXA-48-like (class D) [11]. These genes are commonly associated with mobile genetic elements, located on plasmids that facilitate their dissemination through vertical or horizontal transfer [12]. Plasmids also often carry additional genes for non-β-lactam antibiotic resistance, including aminoglycosides (e.g., 16S rRNA methyltransferases) and plasmid-mediated quinolone resistance, leading to further limitation of treatment options for carbapenemase-producing Enterobacterales (CPEs) infections [13].

Different types of plasmids are involved in the transfer of carbapenemases, among which IncF, IncL/M, IncA/C, and IncX are the most abundant in the *Enterobacteriaceae* family [14]. IncF plasmids, for example, are globally distributed and carry carbapenemases such as KPC and NDM, as well as ESBL enzymes, in particular CTX-M-15. IncX plasmids, especially the IncX3 variant, play a major role in the dissemination of NDM carbapenemases. IncL/M plasmids are also associated with the transmission of NDM among members of the family and can also carry OXA-48. IncA/C plasmids are involved in the dissemination of NDM, VIM, and KPC carbapenemases as well as of cephalosporinases (e.g., CMY) [15].

In Bulgaria, CPE isolates were detected as early as 2007 [16]. Various clinical cases involving KPC-, VIM-, OXA-48-, and NDM-producing strains of *Klebsiella pneumoniae*, as well as an outbreak of NDM-positive *Escherichia coli*, were documented up until 2014 [17]. Since then, the incidence of infections caused by *K. pneumoniae* strains producing NDM-, KPC-, and OXA-48-like enzymes has been steadily rising [11]. However, the role of the CP-ESCPM group remains uncertain, as their reporting is sporadic compared to other CPEs.

This study represents a retrospective genomic analysis of carbapenemase-producing ESCPM isolates collected in Bulgaria between 2014 and 2023. Through a combination of whole-genome sequencing (WGS) and conjugation experiments, our investigation provides insights into the genetic diversity of CP-ESCPM and the plasmids involved in the dissemination of carbapenemases and the associated resistance genes.

## 2. Results

### 2.1. Exploring Carbapenemase Diversity and Plasmid-Mediated Resistance in ESCPM Isolates

Carbapenemase genes were detected in all 36 carbapenemase-producing isolates, with the majority containing bla_VIM_ and/or bla_NDM_ metallo-β-lactamases. Notably, carbapenemases from all the detected families were present in *S. marcescens* and *C. freundii* (Table 1).

**Figure 1:**
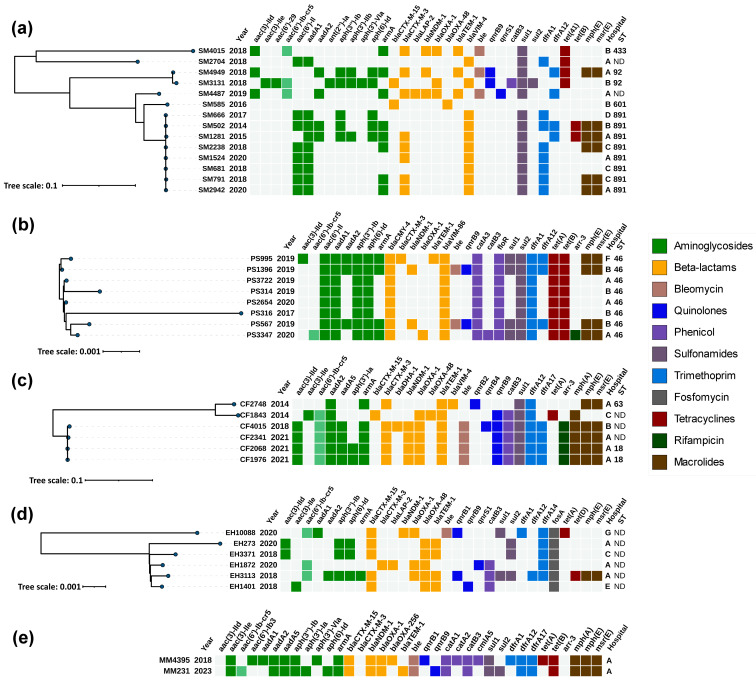
Heatmap representation of the acquired antimicrobial resistance genes of (**a**) *Serratia marcescens*; (**b**) *Providentia stuartii*; (**c**) *Citrobacter freundii*; (**d**) *Enterobacter hormaechei*; (**e**) *Morganella morganii*. Phylogenetic trees were constructed based on SNP analysis for (**a**–**d**) with PhaME v1.0.4 (see Section 4.5). There were only two *M. morganii* isolates, hence the lack of dendrogram. Drug classes are colored consistently, as shown in the legend (right). The only exception from the legend is *aac(6′)-Ib-cr5*, colored in pale green to indicate dual resistance. Both dendrograms and heatmaps were displayed and annotated with iTOL v6.8.1 (https://itol.embl.de/about.cgi, accessed on 3 April 2024). Sequence type (ST) was derived for the species with available MLST schemes; ND, not determined either due to missing alleles or imperfect match. SM, *Serratia marcescens*; PS, *Providentia stuartii*; CF, *Citrobacter freundii*; EH, *Enterobacter hormaechei*; MM, *Morganella morganii*.

The carbapenemases and their alleles were then confirmed by WGS. They were accompanied by a wide range of plasmid-mediated and chromosome-encoded resistance genes. The most prevalent were the VIM alleles *bla*_VIM-4_ (n = 11) and *bla*_VIM-86_ (n = 8), found in a total of 19/36 isolates. *bla*_NDM-1_ was identified in 12/36 and *bla*_OXA-48_ in 7/36 isolates. Interestingly, *bla*_VIM-86_ was identified only in *P. stuartii* isolates, and two of them also co-harbored an additional *bla*_NDM-1_ gene. Figure 1 illustrates the correlation among acquired antimicrobial resistance genes, the year and hospital of isolation, MLST profiles (where applicable), as well as the phylogenetic relatedness of isolates within each analyzed species.

All *bla*_VIM-86_ and *bla*_OXA-48_ genes, as well as all but two *bla*_NDM-1_ genes, were successfully transferred by conjugation, confirming their plasmid origin (Table 2). A transconjugant was also obtained for the *bla*_VIM-4_-positive *C. freundii* isolate. The localization of carbapenemase genes in isolates which failed to transfer their plasmids was inferred from WGS data.

#### 2.1.1. *Serratia marcescens*

We identified a cluster (ST891) of eight epidemiologically unrelated *S. marcescens* isolates from four hospitals between 2014 and 2020 (Figure 1a and Table 2). This cluster was characterized by a non-transferable VIM-4 along with ArmA and CTX-M-3 on IncM2 plasmid detected in 50% of the isolates. The latter plasmid was similar to the previously described pCTX-M3 [18]. The remaining two VIM-4 positive isolates lacked ArmA and CTX-M-3 and were distant from the VIM-4 cluster. Multiple transfer attempts of VIM-4 failed, and BLAST analysis of the upstream and downstream sequences revealed homology with various chromosomal sequences, suggesting chromosomal origin.

Next, close to the VIM-4 cluster was the SM585 isolate, with a chromosomal CTX-M-15 and IncL plasmid with OXA-48. The last three isolates (SM4015, SM4949, and SM4487) were NDM-positive and shared an IncM2 plasmid carrying CTX-M-3 and ArmA similar to the VIM-4 cluster. Interestingly, the genetic context of NDM-1 seems to differ among those three isolates. According to the mating-out experiments, in SM4949, NDM-1 was situated on the conjugative IncM2 plasmid along with CTX-M-3/ArmA, whereas in SM4487, the BLAST analysis of the surrounding sequences of NDM-1 suggested its chromosomal localization, consistent with the failed mating attempts. In SM4015, an IncX plasmid was identified as the NDM-1 carrier, which was confirmed by PCR.

#### 2.1.2. *Providentia stuartii*

*P. stuartii* isolates were collected between 2017 and 2020 and all belonged to ST46. They exhibited close genetic relatedness and consistently harbored an IncC plasmid, with one of the isolates having an additional IncM2 plasmid (Figure 1b and Table 2). Successful transfer of both plasmids was achieved for all studied *P. stuartii* isolates and the PCR replicon typing confirmed the presence of IncA/C replicons in all of the transconjugants and the IncL/M replicon in TC–PS995/2. 

The IncC transconjugants could be divided into three groups based on plasmid transfer and their acquired resistance determinants. The first group of two transconjugants (TC–PS567 and TC–PS1396) harbored both NDM-1 and the recently identified VIM-86 [19], indicative of their co-localization on the same plasmid. Additionally, this plasmid was positive for ArmA, QnrB, and CMY-4. The IncC plasmid of the second group of five transconjugants was associated with VIM-86 and CMY-4. Thirdly, TC-PS3347 demonstrated the transfer of IncC with OXA-1 and AAC(6′)-Ib-cr5 alongside VIM-86, CMY-4, and ArmA.

Lastly, the IncM2 plasmid, carrying only CTX-M-3 and ArmA, initially found to be co-hosted by PS995 together with another IncC plasmid, was successfully conjugated through the mating-out experiments.

#### 2.1.3. *Citrobacter freundii*

For *C. freundii* isolates, one distinct cluster was identified (Figure 1c and Table 2). The cluster consisted of four NDM-1 producers, which included CF4015 (Hospital B) from 2018 and three epidemiologically related isolates from 2021—CF2341, CF2068, and CF1976 (Hospital A). WGS identified Col, IncFIB, IncM2, and IncR replicons in all four isolates. They showed a similar resistance profile, but CF4015 also carried the QnrB4 and DHA-1 genes, while CF2068 and CF1976 carried aph(3′)-Ia. Their IncM2 plasmid was successfully transferred, yielding transconjugants positive for NDM-1, ArmA, CTX-M-3, and QnrB, similar to IncM2 from SM4949, suggesting the interspecies dissemination of this plasmid.

The remaining isolates were substantially different from each other. CF2747 carried VIM-4 on an IncM2 plasmid together with ArmA and CTX-M-3, while CF1843 harbored only OXA-48 on an IncL plasmid. Both plasmids were successfully transferred and the replicons were confirmed by PCR. The OXA-48-encoding IncL plasmid in the CF1843 genome appeared to be similar to the plasmids observed in the SM585, EH273, EH3371, and EH1401 isolates. In TC–CF2747, VIM-4 was transferred along with ArmA and CTX-M-3, as confirmed by PCR.

#### 2.1.4. *Enterobacter hormaechei*

*E. hormaechei* isolates were mainly associated with OXA-48 (Figure 1d and Table 2). The only exception was EH10088, in which we identified NDM-1 and only a Col replicon. Unfortunately, the latter was not confirmed in the corresponding transconjugant by PCR and this led to the assumption that another non-typeable plasmid or an integrative conjugative element might be involved.

The OXA-48 cluster was formed by isolates collected between 2018 and 2020. All donors from that cluster were able to transfer OXA-48. IncL/M replicons were confirmed in all of them, and the in silico BLAST analysis of donor sequences further confirmed the IncL plasmid as the host of the OXA-48 gene.

In one transconjugant, TC–EH1872, OXA-48 was co-transferred with CTX-M-3 (Table 2). In TC–EH3113, OXA-48 was transferred along with ArmA, OXA-1, AAC(6′)-Ib-cr, and QnrB, and PCR detected only the IncL/M replicon. However, BLAST analysis of donor sequences failed to confirm the co-localization of all those genes within the IncL replicon due to fragmented assembly. In conclusion, we are uncertain that the transconjugant has received a single or more plasmids, because PCR-based replicon typing may also have failed to identify more types.

#### 2.1.5. *Morganella morganii*

Two epidemiologically unrelated *M. morganii* isolates recovered from one hospital (A) were identified as producers of NDM-1 (Figure 1e).

Carbapenemase was transferred only in TC–MM4395, suggesting that the gene is encoded by a conjugative IncT plasmid, as this was the only replicon found in the donor. The co-transfer of additional resistance genes, including ArmA, OXA-1, AAC(6′)-Ib, and QnrB, was also confirmed by PCR. Despite the presence of the IncT replicon in the donor, PCR assay failed to confirm it in the transconjugant (Table 2). However, aligning the reads to closely related plasmids provided strong evidence that NDM-1 and the other resistance genes are encoded on IncT plasmid.

In contrast, no plasmid replicons were detected in MM231, and it failed to transfer any resistance determinants through conjugation, implying the chromosomal location of NDM-1 and the associated resistance determinants.

### 2.2. Antibiotic Susceptibility

The results from the susceptibility testing showed that all isolates were resistant to penicillins, penicillin/inhibitor combinations, cephalosporins, cephamycins, ertapenem, doripenem, quinolones, and tobramycin, but showed variable results for imipenem, meropenem, ceftazidime-avibactam, aztreonam, amikacin, gentamicin, colistin, tigecycline, fosfomycin, trimethoprim–sulfamethoxazole, and cefiderocol. The distribution of MIC values is shown in Supplementary Table S1.

Disc susceptibility testing revealed that 78% (28/36) of all isolates were susceptible to cefiderocol. This included all isolates of ESCM species and excluded the isolates of *P. stuartii* as they exhibited resistance. Across all isolates, cefiderocol had the highest activity among all antimicrobials tested, followed by amikacin with 42% (15/36) susceptibility. Colistin and tigecycline were effective only against *C. freundii* and *E. hormaechei*, which comprised 33% (12/36) of the total isolates. Ceftazidime–avibactam demonstrated in vitro activity limited to OXA-48 producers, accounting for 19% (7/36) of all isolates. The other antimicrobial agents displayed varying levels of activity against ESCPM isolates, necessitating individual treatment regimens with combinations of antibiotics from different classes [4,20].

## 3. Discussion

In Bulgaria, carbapenem-non-susceptible *Enterobacterales* isolates are optionally referred to the National Reference Laboratory for Control and Monitoring of Antimicrobial Resistance for verification. Apart from *K. pneumoniae*, the nationwide frequency of ESCPM isolates reporting and referral remains unknown. We therefore hypothesized that they persist in many hospitals and that their actual numbers, and consequently their impact on human health, are underestimated. To address this issue, thirty-six carbapenemase-positive ESCPM isolates were subjected to WGS analysis and mating-out experiments to determine their plasmid content and resistance transferability.

Most of the carbapenemase-producing ESCPM isolates originated from urine (59.5%) and wound specimens (27.0%), and only 5.4% from blood. The origin of ESCPM pathogens reflects their ability to cause various nosocomial infections, mainly those of the urinary tract as well as intra-abdominal and soft tissue infections, but also bacteremia and respiratory tract infections [2].

In this study, the majority of carbapenemases within the ESCPM group were found on conjugative plasmids. Plasmids belonging to the IncL/M group were most commonly identified among the obtained transconjugants. Originally considered a single plasmid incompatibility group, IncL/M plasmids underwent re-evaluation and were subsequently categorized into two separate groups: IncL and IncM [21].

IncL plasmids have been associated with the global dissemination of the OXA-48 gene, while IncM2 plasmids have been linked to the spread of NDM-1 [21]. Consistent with these observations, our study revealed a strict association between the presence of IncL-type plasmids and the carriage of OXA-48. In the majority of transconjugants, OXA-48 was the sole resistance gene transferred. But instances of co-transfer were also observed, including the transfer of OXA-48 alongside CTX-M-3 in TC–EH1872 and the transfer of ArmA, OXA-1, AAC(6′)-Ib-cr, and QnrB in TC–EH3113 (Table 2). However, the genetic landscape of the detected additional genes remained ambiguous in some cases as the sequences appeared fragmented, probably due to the presence of mobile genetic elements.

IncM2 plasmids are known to harbor genes conferring resistance to various antimicrobial agents, including cephalosporins, carbapenems, aminoglycosides, trimethoprim, sulphonamides, and fosfomycin [22]. In this study, they were mainly involved in NDM-1 and VIM-4 dissemination among isolates of *C. freundii* (n = 5) and *S. marcescens* (n = 1) (Table 2). We observed a consistent pattern of co-transfer of CTX-M-3 and ArmA along with carbapenemases through a plasmid with a similar genetic content to the original pCTX-M-3 plasmid [18].

In the two *P. stuartii* isolates (PS1396 and PS567) harboring both NDM-1 and VIM-86 carbapenemases, we found multiple resistance determinants. Using long-read sequencing, we were able to reconstruct the entire IncC plasmid of PS1396, revealing that the majority of resistance genes (CMY-4, ArmA, VIM-86, NDM-1, and QnrB9) are co-localized within it. Likewise, in PS3347, we identified a similar IncC plasmid bearing ArmA, VIM-86, OXA-1, and AAC(6′)-Ib-cr5. VIM-86 was recently described by Rezzoug and colleagues [19]. The structure of the plasmid hosting this novel allele closely resembles that of the PS1396 plasmid, albeit lacking the NDM-1 gene. While not explored in this study, determining the origin of NDM-1 in the PS1396 plasmid is expected to be a primary focus of our future investigations.

For isolate SM4015, we obtained two distinct transconjugants (TC–SM4015/1 and TC–SM4015/2). TC–SM4015/1 carried an IncX3 plasmid encoding NDM-1, while TC–SM4015/2 harbored an IncM2 plasmid of the pCTX-M-3 type. IncX3 plasmids, known for their narrow host range, have been associated with the dissemination of various NDM variants among *Enterobacteriaceae* [23]. Their significance as vectors for clinically relevant antibiotic resistance genes has been increasingly recognized in recent years [24,25].

In one isolate (MM4395), NDM-1 was detected alongside ArmA, possibly situated on an IncT-like plasmid. However, we were unable to confirm this since only PCR was employed for the screening of transconjugants, and the assay failed to detect any plasmid replicons in the respective transconjugant. Nevertheless, our investigation using BLAST searches with the sequence containing NDM-1 identified a pZ26CR2253_NDM IncT-type plasmid from *Providencia huaxiensis* (CP145930.1). Mapping the raw reads to this plasmid revealed substantial 94% template coverage, providing strong evidence for the association of the NDM-1 gene with the donor IncT plasmid. IncT plasmids have a narrow host range and are rarely reported. Previous studies have identified IncT plasmids in various species such as *Proteus mirabilis*, *C. freundii*, and *Providencia rettgeri*, where they were associated with ESBLs (CTX-M-2) [26] and carbapenemases of class D (OXA-181) and class B (NDM-1) [27].

In the present study, all of the identified carbapenemase types were found in *S. marcescens* and *C. freundii*. In *S. marcescens* isolates VIM-4 was localized on the chromosome. NDM-1 was encoded mainly on plasmids and was detected among all of the tested species. VIM-type carbapenemases were mostly detected in *P. stuartii* and *S. marcescens*, while OXA-48-like genes were predominantly found in *E. hormaechei*. These findings are consistent with the results of a recent surveillance study of 6774 ESCPM blood culture isolates from 27 European hospitals [20]. 

A significant concern was the detection of ArmA methyltransferase in association with carbapenemases in CP-ESCPM with intrinsic resistance to colistin and tigecycline (such as *P. stuartii* and *M. morganii*). As ArmA confers high-level resistance to clinically relevant aminoglycosides, infections caused by these pathogens present a serious treatment challenge, as there are almost no treatment options left.

There are a few limitations to this study. First, we did not perform WGS for resulting transconjugants, which could improve the detection and typing of the received plasmids and their exact gene content. Next, the assembly of plasmids is often difficult and incomplete with short-read data only. Consequently, the genetic environment of the detected genes was not examined further. Including long-read data from donor isolates and/or transconjugants will drastically enhance the quality of the assembled genomes and thus the plasmid analysis. Finally, we included a relatively small number of isolates per studied species. Routine WGS molecular surveillance is still not implemented in Bulgaria, but increasing the sequencing capacity will provide more reliable and broader understanding of the plasmid diversity and resistance genes of the ESCPM group. 

## 4. Materials and Methods

### 4.1. Bacterial Isolates

Thirty-six carbapenem-non-susceptible ESCPM isolates were sent by seven hospital laboratories to the National Reference Laboratory for Control and Monitoring of Antimicrobial Resistance in Bulgaria between July 2014 and January 2023 for molecular validation of carbapenem resistance. The isolates were re-identified using the MALDI-TOF Biotyper (Bruker Daltonics GmbH, Bremen, Germany) with MALDI Reference 2022 Library v.4.0. The studied ESCPM group comprised *S. marcescens* (n = 14), *P. stuartii* (n = 8), *C. freundii* (n = 6), *E. hormaechei* (n = 6), and *M. morganii* (n = 2) isolates. They were anonymized and were assigned code names (e.g., MM4395 for *Morganella morganii* strain number 4395).

All carbapenem-non-susceptible ESCPM isolates were confirmed as carbapenemase producers by CarbaNP test [28]. Sources and years of isolation are summarized in Supplementary Table S2 and Supplementary Table S3, respectively. Hospital laboratories were named with capital letters from A to G depending on the number of the submitted isolates (Supplementary Table S4).

### 4.2. Antimicrobial Susceptibility Testing and Detection of Carbapenemases

Disc susceptibility testing was performed according to EUCAST guidelines [29] on Mueller–Hinton agar with disks supplied by Becton Dickinson (BD, Sparks, MD, USA). MICs were determined by broth microdilution using the MicroScan NM-EN52 panel (Beckman Coulter, Inc., Brea, CA, USA) and the Micronaut-S MDR plate (Merlin Diagnostika GmbH, Bornheim, Germany) by following the manufacturer’s protocols. Susceptibility testing results were interpreted in accordance with EUCAST clinical breakpoints v13.0. *E. coli* ATCC 25922 was used for quality control.

### 4.3. Screening for Carbapenemase Genes

Total genomic DNA for PCR and whole-genome sequencing was extracted using the PureLink™ Genomic DNA Mini Kit (Thermo Fisher Scientific, Missouri, TX, USA) following the manufacturer’s instruction, except that all homogenization steps were performed by pipetting. Primer pairs from prior publications were used in a previously described multiplex PCR [30] to screen all isolates for class A, class B, and class D carbapenemases [31,32,33,34,35]. PCR conditions are detailed in Supplementary Table S5.

### 4.4. Whole-Genome Sequencing

All isolates that were confirmed to carry carbapenemase genes were subjected to short-read sequencing with an Illumina DNA Prep kit (Illumina, San Diego, CA, USA) with MiSeq V3 (2 × 300 bp) or NextSeq 550 with V2.5 (2 × 150 bp) mid-output flow cell (Illumina, Inc., San Diego, CA, USA). The same DNA extracts of PS1396 and PS995 were also sequenced on a MinION Mk1C with the Rapid Barcoding Kit 96 (SQK-RBK110.96) and FLO-MIN106D (R9.4.1) (Oxford Nanopore Technologies, Oxford, UK) with a slight modification. The final size selection of the library pool was carried out using 0.4x SPRI magnetic particles to eliminate fragments < 1.5 kb [36].

### 4.5. Bioinformatic Analysis

The quality of the raw reads was assessed using FastQC v0.11.9 (https://www.bioinformatics.babraham.ac.uk/projects/fastqc, accessed on 3 April 2024). Subsequent quality trimming and filtering for short and long reads were carried out using fastp v0.23.2 [37] and filtlong v0.2.1 (https://github.com/rrwick/Filtlong, accessed on 3 April 2024). Short-read assemblies were generated with Unicycler v0.4.8 [38]. For PS1396 and PS995, a long-read-only assembly was produced using Trycycler v0.5.3 [39] followed by polishing with MEDAKA v1.7.3 (ONT, https://github.com/nanoporetech/medaka, accessed on 3 April 2024). KmerFinder v3.0.2 [40] with a database version from 11 July 2022 was used for in silico identification. Assemblies were annotated using Bakta v 1.7 [41] with database v5.0-full. Screening for genes related to antimicrobial resistance and prediction of phenotypes were carried out using AMRFinderPlus v3.11.4 [42] and ResFinder v4.3.1 [43] (database v2022-05-24). The sequences containing carbapenemases were further analyzed with BLAST using the nr/nt database (Update date: 19 March 2024). Plasmid analysis was conducted using Abricate (Seemann T, Abricate, Github, San Francisco, CA, USA, https://github.com/tseemann/abricate, accessed on 3 April 2024) with the PlasmidFinder database (v2023-01-18) [44]. In silico MLST profiling was performed with mlst v2.23.0 (Seemann T, mlst Github, https://github.com/tseemann/mlst, accessed on 3 April 2024).

Phylogenies for individual species were constructed using PhaME v1.0.4 [45], considering only SNPs within the coding regions of the core genome. The resulting maximum-likelihood trees were linked to antimicrobial resistance patterns and visualized with iTOL [46].

### 4.6. Transfer of Resistance Determinants and Identification of Transconjugant Plasmids

Transfer of carbapenem and 16S rRNA methyltransferase-mediated aminoglycoside resistance was attempted by mating on filters with sodium azide-resistant *E. coli* J53 recipient strain as previously described [47]. Transconjugants were selected on MacConkey agar containing sodium azide (100 mg/L) supplemented with either meropenem (0.5 mg/L) or amikacin (50 mg/L) and gentamicin (50 mg/L). Transconjugants were confirmed by susceptibility testing and PCR. Detection of carbapenemase genes was performed as described for the donor strains. Other co-transferred antibiotic resistance genes were detected by various multiplex PCRs. CTX-M genes were detected as described previously [48]. OXA-1/2/9/10-like genes [49] were screened with a protocol listed in Supplementary Table S6. Plasmid-mediated AmpC beta-lactamase genes were detected as previously published [50]. Identification of 16S rRNA methyltransferase genes (*armA*, *rmtB*, *npmA*, *rmtA*, *rmtC*, *rmtD*, *rmtE*, *rmtF*) [51,52,53] was carried out as described in Supplementary Table S7, whereas plasmid-mediated quinolone resistance determinants (*qnrA*, *qnrB*, *qnrC*, *qnrD*, *qnrS*, *qepA*) [54,55,56,57] were detected as described in Supplementary Table S8. Lastly, the detection of *aac(6′)-Ib-cr* acetyltransferase was performed as described previously [58].

Replicon typing involved a combination of multiplex and singleplex PCR panels targeting 21 plasmid replicons as detailed in a previous study [59]. All amplicons were visualized on QIAxcel Advanced high-resolution capillary electrophoresis system (Qiagen, Hilden, Germany) with protocol 0M800 for precise size estimation.

## 5. Conclusions

In this study, we analyzed the genetic characteristics of CP-ESCPM species isolated between 2014 and 2023 in Bulgaria, with an emphasis on the dissemination of carbapenemases and the significance of the self-conjugative plasmids in the transmission of resistance determinants. The predominant carbapenemase was VIM, represented by VIM-4 (n = 11) and VIM-86 (n = 8), found in a total of 19/36 isolates. NDM-1 was detected in 12/36 and OXA-48 in 7/36 isolates. VIM-86 was identified only in *P. stuartii* isolates, two of which also carried NDM-1. IncL plasmids were responsible for the spread of OXA-48 among *E. hormaechei*, *C. freundii*, and *S. marcescens*. IncM2 plasmids were generally associated with the spread of NDM-1 in both *C. freundii* and *S. marcescens*, and also of VIM-4 in *C. freundii*. IncC plasmids have been implicated in the spread of the recently described VIM-86 in *P. stuartii* isolates. IncC plasmids carrying NDM-1 and VIM-86 have also been observed. NDM-1 was detected on IncX3 in *S. marcescens* and on IncT plasmid in *M. morganii*. The diversity of CP-ESCPM and carbapenemase-encoding plasmids that were identified in this study highlights the role of the ESCPM group as a reservoir of resistance determinants in hospital settings. Our results stress the need to strengthen control and implement surveillance measures for the carbapenemase-producing ESCPM to limit the spread of antibiotic resistance.

## Figures and Tables

**Table 1:** Distribution of carbapenemase genes among the 36 CP-ESCPM isolates.

Species	VIM	NDM	VIM + NDM	OXA-48-like	Total
*S. marcescens*	10	3		1	14
*P. stuartii*	6		2		8
*C. freundii*	1	4		1	6
*E. hormaechei*		1		5	6
*M. morganii*		2			2
Total	17 (47.2%)	10 (27.8%)	2 (5.6%)	7 (19.4%)	36

**Table 2:** Characteristics of transconjugants and donor plasmid replicon types.

Transconjugant	Transferred Resistance Genes and Plasmid Incompatibility Group			Associated Phenotypic Resistance ^a^	Donor Plasmid Replicon Type(s) ^b^ (WGS)
	Carbapenemase Gene(s)	Additional Resistance Genes	Inc Group (PCR)		
TC—SM4015/1	*bla*_NDM-1_	-	IncX	-	**IncX3**; IncM2
TC—SM4015/2	*-*	*armA*, *bla*_CTX-M-3_, *bla*_OXA-1_, *aac(6′)-Ib-cr*	IncL/M	-	**IncM2**; IncX3
TC—SM4949	*bla*_NDM-1_	*armA*, *bla*_CTX-M-3_, *qnrB*	IncL/M	Sxt	IncM2
TC—SM4487	*-*	*armA*, *bla*_CTX-M-3_	IncL/M	Sxt	IncM2; Col
TC—SM585	*bla*_OXA-48_	-	IncL/M	-	**IncL**
TC—SM502	-	*armA*	ND	-	IncFII; IncHI2; IncHI2A; Col
TC—SM1281	-	*armA*, *bla*_CTX-M-3_	IncL/M	Sxt	**IncM2**; IncFII; IncHI2; IncHI2A CCol
TC—SM2238	-	*armA*, *bla*_CTX-M-3_	IncL/M	-	**IncM2**; IncFII
TC—SM791	-	*armA*, *bla*_CTX-M-3_	IncL/M	-	**IncM2**; IncFII
TC—SM2942	-	*armA*, *bla*_CTX-M-3_	IncL/M	-	**IncM2**; IncFII; Col
TC—PS995/1	*bla*_VIM-86_	*bla*_CMY-4_	IncA/C	Sxt Cm Te Tm	**IncC**; IncM2
TC—PS995/2	-	*armA*, *bla*_CTX-M-3_	IncL/M	Sxt	**IncM2**; IncC
TC—PS1396	*bla*_VIM-86_, *bla*_NDM-1_	*armA*, *bla*_CMY-4_, *qnrB*	IncA/C	Sxt Cm Te	**IncC**
TC—PS3722	*bla*_VIM-86_	*bla*_CMY-4_	IncA/C	Sxt Cm Te Tm	**IncC**
TC—PS314	*bla*_VIM-86_	*bla*_CMY-4_	IncA/C	Sxt Cm Te Tm	**IncC**
TC—PS2654	*bla*_VIM-86_	*bla*_CMY-4_	IncA/C	Sxt Cm Te Tm	**IncC**
TC—PS316	*bla*_VIM-86_	*bla*_CMY-4_	IncA/C	Sxt Cm Te Tm	**IncC**
TC—PS567	*bla*_VIM-86_, *bla*_NDM-1_	*armA*, *bla*_CMY-4_, *qnrB*	IncA/C	Sxt Cm Te	**IncC**
TC—PS3347	*bla*_VIM-86_	*armA*, *bla*_CMY-4_, *bla*_OXA-1_, *aac(6′)-Ib-cr*	IncA/C	Sxt Cm Te	**IncC**
TC—CF2748	*bla*_VIM-4_	*armA, bla*_CTX-M-3_	IncL/M	Sxt	**IncM2**; IncFIB; IncFII; Col
TC—CF1843	*bla*_OXA-48_	-	IncL/M	-	**IncL**; IncFII; IncFIA; Col
TC—CF4015	*bla*_NDM-1_	*armA*, *bla*_CTX-M-3_, *qnrB*	IncL/M	Sxt	**IncM2**; IncFIB; IncR;Col
TC—CF2341	*bla*_NDM-1_	*armA*, *bla*_CTX-M-3_, *qnrB*	IncL/M	Sxt	**IncM2**; IncFIB; IncR;Col
TC—CF2068	*bla*_NDM-1_	*armA*, *bla*_CTX-M-3_, *qnrB*	IncL/M	Sxt	**IncM2**; IncFIB; IncR;Col
TC—CF1976	*bla*_NDM-1_	*armA*, *bla*_CTX-M-3_, *qnrB*	IncL/M	Sxt	**IncM2**; IncFIB; IncR;Col
TC—EH10088	*bla*_NDM-1_	-	ND	-	Col
TC—EH273	*bla*_OXA-48_	-	IncL/M	-	**IncL**; IncFIB; IncX5; Col
TC—EH3371	*bla*_OXA-48_	-	IncL/M	-	**IncL**; IncFII; IncFIB; Col
TC—EH1872	*bla*_OXA-48_	*bla*_CTX-M-3_	IncL/M	-	**IncL**; IncFII; Col
TC—EH3113	*bla*_OXA-48_	*armA*, *bla*_OXA-1_, *aac(6′)-Ib-cr, qnrB*	IncL/M	Sxt	**IncL**; IncFII; IncFIB; Col
TC—EH1401	*bla*_OXA-48_	-	IncL/M	-	**IncL**; IncFII; IncFIB; IncX3
TC—MM4395	*bla*_NDM-1_	*armA*, *bla*_OXA-1_, *aac(6′)-Ib, qnrB*	ND	Sxt Cm	IncT

TC, transconjugant; SM, Serratia marcescens; PS, Providentia stuartii; CF, Citrobacter freundii; EH, Enterobacter hormaechei; MM, Morganella morganii; “-”, absence of gene or resistance; ND, not determined. ^a^ Sxt, trimethoprim-sulfamethoxazole; Cm, chloramphenicol; Te, tetracycline; Tm, tobramycin. ^b^ The replicon type of donor plasmid in bold is that corresponding to the incompatibility group of the transconjugant plasmid identified by PCR-based replicon typing.

## Data Availability

All data generated during the study are included in this article and its Supplementary Materials. Relevant links and references to other sources are included in the main text. The draft genome sequences were uploaded to the National Center for Biotechnology Information (NCBI) database and are accessible through BioProject ID PRJNA1077246, except for the complete genome sequences of *P. stuartii* isolates PS1396 and PS995, deposited in the European Nucleotide Archive (ENA) under project accession number PRJEB70804.

## Supplementary Materials

The following supporting information can be downloaded at: https://www.mdpi.com/article/10.3390/antibiotics13050455/s1, Table S1: MIC distributions for carbapenemase-positive ESCPM isolates; Table S2: Sources of the 36 carbapenemase-producing ESCPM isolates; Table S3: Distribution of 36 carbapenemase producers according to year of isolation; Table S4: Distribution of 36 carbapenemase producers according to submitting hospital; Table S5: Carbapenemase mPCR; Table S6: OXA mPCR; Table S7: 16S rRNA methyltransferase (16S-RMTase) mPCR; Table S8: Plasmid-mediated quinolone resistance (PMQR) mPCR.
